# Abstract of Meteorological Observations for March, 1854, Made at Philadelphia, Pa.

**Published:** 1854-05

**Authors:** James A. Kirkpatrick


*Abstract of Meteorological Observations for March,* 1854, *made at Philadelphia, Pa. By* PROF. JAMES A. KIRKPATRICK.

| 1854. March. | BAROMETER. Daily Mean. | THERMOMETER. Daily Mean. | THERMOMETER. Daily Range. | Dew point. 2 P. M. | Prevailing Winds. | General Remarks. |
|---|---|---|---|---|---|---|
| | Inches. | deg. | deg. | deg. | Points. | |
| 1 | 30.148 | 38.5 | 13 | 31.0 | (Var.) | M. cloudy, aft. and ev. clear. |
| 2 | 30.093 | 43.0 | 16 | 39.3 | N.E. | Aft. drizzling; *Rain* .018 in. |
| 3 | 29.749 | 50.0 | 14 | 43.0 | N.N.E. | *Rain* 8 to 9 A.M., .042 in. |
| 4 | 29.743 | 49.5 | 9 | 34.0 | S.W. | M. and ev. clear, aft. cloudy. |
| 5 | | | | | | Observations missed. |
| 6 | 30.100 | 40.0 | 14 | 28.3 | N.W. | M. and aft. clear, ev. cloudy. |
| 7 | 29.893 | 46.0 | 13½ | 45.0 | (Var.) | Cloudy. |
| 8 | 29.488 | 52.5 | 9 | 52.3 | N.E. | *Rain* 6½ to 10½ A.M., .256 in. ev. clear. |
| 9 | 29.685 | 47.5 | 5 | 45.3 | E.N.E | *Rain* 9 A.M. till night, .373 in. |
| 10 | 29.507 | 51.5 | 17 | 57.7 | N.E. | Cloudy, ev. *Rain*, .255 in. |
| 11 | 29.940 | 47.0 | 8 | 32.3 | N. | M. & aft. cloudy, ev. clear. |
| 12 | 30.153 | 46.0 | 20 | 35.0 | S.W. | Clear. |
| 13 | 29.981 | 52.0 | 24 | 40.7 | S.W. | Cloudy. |
| 14 | 29.779 | 60.5 | 19 | 46.7 | S.W. | M. cloudy, aft. & ev. clear. |
| 15 | 29.763 | 58.5 | 15 | 45.0 | S.W. | Cloudy. |
| 16 | 29.393 | 65.5 | 19 | 44.7 | S.W. | M. and aft. cloudy, ev. clear; *Rain* 4¼ to 4¾ P.M., .003 in. Therm. highest 75 deg. |
| 17 | 29.392 | 55.5 | 17 | 29.0 | S.W. | M. clear, aft. & ev. cloudy; ev'ng. high wind; Barom. lowest 29.158. |
| 18 | 29.735 | 32.0 | 8 | 24.3 | N.W. | Clear, high wind all day. |
| 19 | 30.063 | 30.0 | 14 | 20.7 | W. | Clear. |
| 20 | 30.044 | 33.0 | 10 | 24.0 | N.W. | M. & ev. clear, aft. cloudy. |
| 21 | 30.174 | 29.5 | 14 | 25.3 | W. | Clear. |
| 22 | 29.878 | 34.0 | 4 | 31.3 | E. | Cloudy, *Rain* all the aft. and till about 5 A.M., .345 in. Therm. lowest 22 deg. |
| 23 | 29.502 | 43.0 | 16 | 41.7 | N.W. | Evening clear. |
| 24 | 29.522 | 37.0 | 10 | 25.7 | (Var.) | M. & aft. cloudy, ev. clear. |
| 25 | 29.629 | 29.0 | 6 | 27.3 | W.N.W. | M. & ev. clear, aft. cloudy. |
| 26 | 29.683 | 32.0 | 10 | 25.3 | N. W. | Clear. |
| 27 | 29.883 | 32.0 | 12 | 17.0 | W.N.W. | Clear. |
| 28 | 30.046 | 28.5 | 7 | 29.7 | N.W. | M. & aft. cloudy, ev. clear. |
| 29 | 30.193 | 30.0 | 14 | 23.0 | N.W. | Clear; Bar. highest 30.260. |
| 30 | 30.131 | 33.0 | 6 | 33.7 | E.N.E. | *Snow* 9¼ A.M till 5½ P.M 112 in. |
| 31 | 29.979 | 40.0 | 6 | 40.7 | N.E. | Drizl'ng *rain* all day .220 in. |
| Monthly Mean 1854 | 29.842 | 42.2 | 53 | 34.6 | NW.SW. | 1.625 inches rain. |
| Monthly Mean 1853 | 29.873 | 41.8 | 53 | 32.7 | WNW.WSW. | 2.460 do. do. |
| Monthly Mean 1852 | 29.900 | 43.1 | 49 | 35.9 | SW.NW. | 4.270 do. do. |
| Monthly mean for 3 years. | 29.872 | 42.4 | 52 | 34.4 | SW.NWW. | 2.785 inches. |

